# Microneedle-mediated drug delivery for cutaneous diseases

**DOI:** 10.3389/fbioe.2022.1032041

**Published:** 2022-10-17

**Authors:** Jian Chen, Hui Ren, Pan Zhou, Shuai Zheng, Bin Du, Xiaowen Liu, Fei Xiao

**Affiliations:** ^1^ Clinical Translational Center for Targeted Drug, Department of Pharmacology, School of Medicine, Jinan University, Guangzhou, China; ^2^ Department of Orthopaedics, The First Affiliated Hospital of Jinan University, Jinan University, Guangzhou, China; ^3^ Department of Pathology, Shanghai First Maternity and Infant Hospital, Tongji University School of Medicine, Shanghai, China

**Keywords:** drug delivery, microneedle, cutaneous disease, transdermal route, microneedle fabrication

## Abstract

Microneedles have garnered significant interest as transdermal drug delivery route owing to the advantages of nonselective loading capacity, minimal invasiveness, simple operation, and good biocompatibility. A number of therapeutics can be loaded into microneedles, including hydrophilic and hydrophobic small molecular drugs, and macromolecular drugs (proteins, mRNA, peptides, vaccines) for treatment of miscellaneous diseases. Microneedles feature with special benefits for cutaneous diseases owing to the direct transdermal delivery of therapeutics to the skin. This review mainly introduces microneedles fabricated with different technologies and transdermal delivery of various therapeutics for cutaneous diseases, such as psoriasis, atopic dermatitis, skin and soft tissue infection, superficial tumors, axillary hyperhidrosis, and plantar warts.

## Introduction

Skin, accounting for 1.2–2.0 square meters of the body and 16% of the bodyweight (Li S. et al., 2020), is the largest organ composed of several layers (epidermis, dermis, subcutis) that protect the body from invasion by foreign substances (Yin and Smith, 2016). These protective layers also provide natural barriers for drugs that are used to treat various deep tissue and dermatologic diseases. The stratum corneum (SC), comprising approximately 15–20 layers of specialized anucleated keratino-cytes, resides within the most superficial layer of the epidermis (Menon et al., 2012), forming a major barrier against the external environment. Therapeutic penetration through this layer is determined by the lipid solubility and molecular weight of the drugs; the transdermal delivery ability of hydrophilic drugs and drugs with a molecular weight higher than 500 Da is very limited (Mishra and Bonde, 2020).

Development of delivery systems that intend to aid penetration of such drugs through deeper layers has been increasing in the past 20 years, extending the applications of these hydrophilic and macromolecule drugs for the treatment of deep tissue and dermatologic diseases. Many physical or pharmaceutical strategies, such as sonophoresis, electroporation, iontophoresis, microneedles (MNs), chemical enhancers, and transdermal formulations, have been developed for transdermal drug delivery (TDD) of these inaccessible hydrophilic and macromolecule drugs, referring to drug administration for local dermatosis or systemic therapy purposes (Pan et al., 2020). These TDD strategies can facilitate the transdermal absorption of a series of hydrophilic or macromolecule drugs, presenting advantages with bypass of gastrointestinal stimuli and degradation, avoidance of first-pass elimination, and improved patient compliance.

**FIGURE 1:**
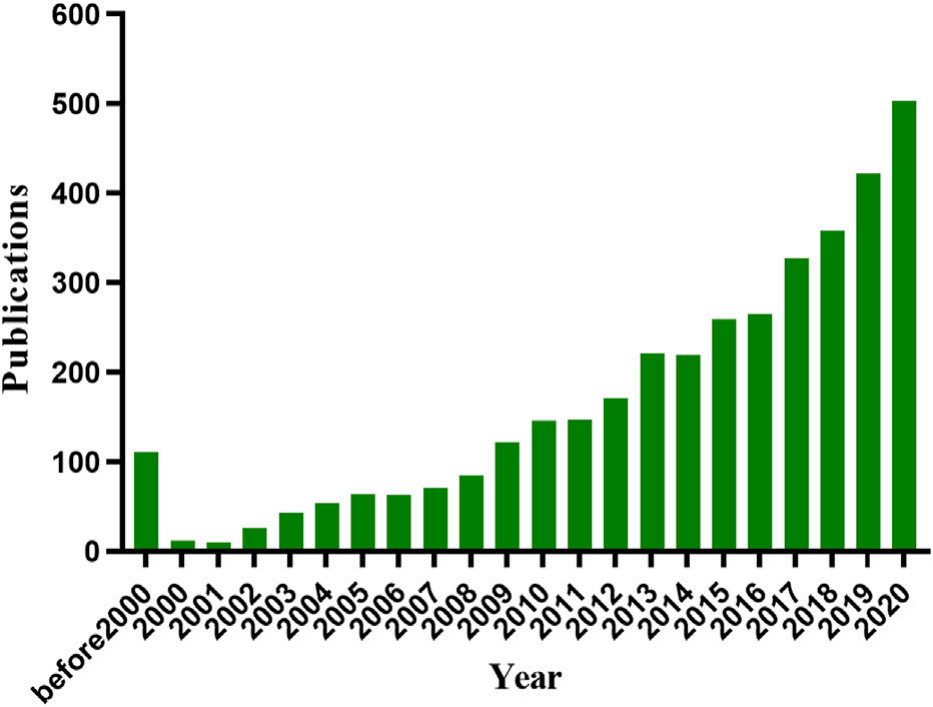
Number of publications on microneedle based on PubMed database (https://www.ncbi.nlm.nih.gov/pubmed/) and Web of Science (http://apps.webofknowledge.com) on 12 November 2021.

Of note, the microneedle-mediated TDD system has gained significant attention, presenting a superior drug delivery route to topical lesions with enhanced transdermal efficiency, microneedles have natural advantages with nonspecific drug loading ability and direct therapeutic delivery route. A number of drugs, including small molecular drugs (Chiang et al., 2016), macromolecular drugs (Prausnitz et al., 2019), vaccines (Waghule et al., 2019), and nano-particles (Du et al., 2017) have been loaded into microneedles to treat various diseases. Drug-loaded microneedles can temporarily break the stratum corneum for enhanced skin permeability and subsequently improve drug penetration efficiency for better therapeutic outcomes. Research in microneedles has been significantly increased in the past 10 years (Figure 1), including manufacturing techniques, microneedle-mediated delivery systems for cancer and diabetes treatment, vaccination, and various skin diseases. Many reviews have summarized advances in the microneedle-mediated delivery system for cancer and diabetes treatment. Accordingly, in this review, we would introduce the applications of the microneedle-mediated drug delivery (MDD) system for treatment of dermatological diseases, including psoriasis, atopic dermatitis, skin and soft tissue infections, superficial tumors, axillary hyperhidrosis, and plantar warts.

## Microneedles

Microneedle, containing a cavity for drug reservoir and a tiny protrusion for transdermal penetration, was first patented in 1971 (Martin and Gerstel, 1971). Since then, microneedles have been extensively developed for TDD along with advances in microfabrication manufacturing and pharmaceutics technologies. According to differences in drug delivery pattern, microneedles can be divided into solid, hollow, dissolved, or coated microneedles (Table 1).

**TABLE 1:** Summary of the main materials, advantages, and disadvantages of various MNs.

MN classification	Materials	Advantages	Disadvantages	References
Solid	Silicon, polylactic acid, stainless steel	Desirable mechanical properties	Low drug loading capacity, limited biological compatibility	Wilke et al. (2005), Narayanan and Raghavan, (2017)
Hollow	Silicon, glass, polymers	High drug loading capacity	Low mechanical strength, fractures	Luttge et al. (2007), Miller et al. (2011)
Dissolving	Maltose, carboxymethylcellulose, hyaluronic acid	Good biocompatibility	Mechanical strength is not high enough	Park and Kim, (2017), Song et al. (2020)
Coated	Silicon, Stainless steel, Polymers	Can be used for potent drugs requiring low doses	Manufacturing process may be contaminated	McGrath et al. (2011), Ita, (2018)

### Solid microneedles

**FIGURE 2:**
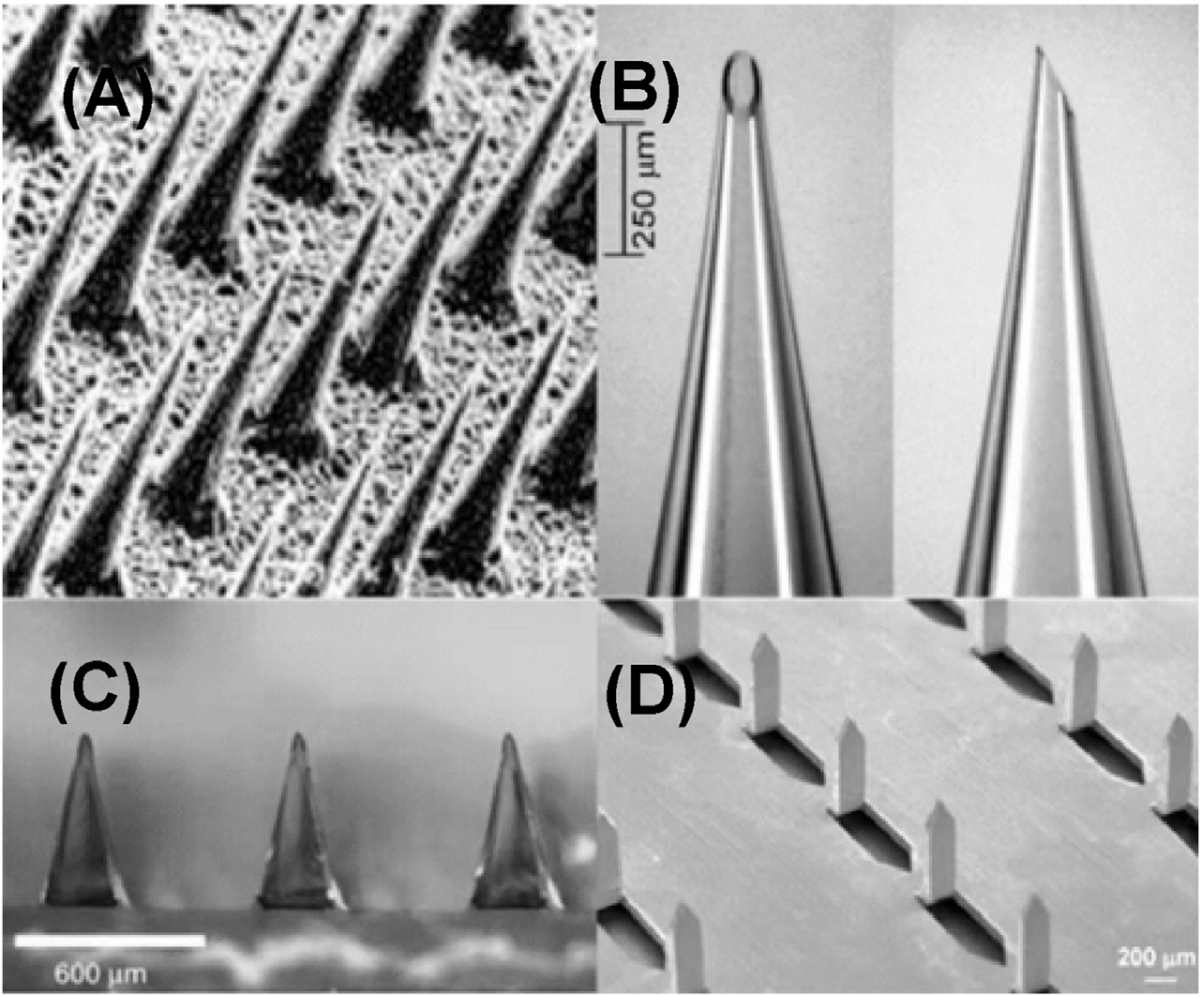
Types of microneedles. **(A)** Solid microneedles. Reproduced with permission (Henry et al., 1998). Copyright 1998, Elsevier. **(B)** Hollow microneedles. Reproduced with permission (Martanto et al., 2006). Copyright 2006, Springer Nature. **(C)** Dissolving microneedles. Reproduced with permission (Lee et al., 2008). Copyright 2008, Elsevier. **(D)** Coated microneedles. Copyright 2007, Springer Nature.

The solid microneedle was typically used for pretreatment of the skin prior to administration of active ingredients (Figure 2A). Common materials used for the manufacture of solid microneedles primarily include silicon (Hashmi et al., 1995; Wilke et al., 2005; Narayanan and Raghavan, 2017, 2019), methyl vinyl ether and maleic anhydride (PMVE/MA) (Donnelly et al., 2011), polymethyl methacrylate (PMMA) (Moon et al., 2005), polylactic acid (PLA) (Li Q. Y. et al., 2017), as well as stainless steel (Martanto et al., 2004), titanium (Matriano et al., 2002), nickel (Jung et al., 2008) and other metallic materials. Solid microneedles can be applied for TDD (Table 2) both with or without drug coating. Non-drug-coated solid microneedles can create transient skin microchannels and enhance drug penetration efficiency for the following topical administration (Li et al., 2017b). Drug-coated solid microneedles function as both skin penetration modules and drug reservoirs. Though rapid delivery of active ingredients can be facilitated using this system, their limitations including low drug loading capacity, limited biological compatibility, and inaccurate dose administration need to be carefully considered (Li et al., 2017a).

**TABLE 2:** Abbreviations table.

Abbreviations	English full name
**SC**	Stratum corneum
**MNs**	Microneedles
**TDD**	Transdermal drug delivery
**MDD**	Microneedle-mediated drug delivery
**PMVE/MA**	Methyl vinyl ether and maleic anhydride
**PMMA**	Polymethyl methacrylate
**PLA**	Polylactic acid
**CyA**	Cyclosporin A
**TA**	Triamcinolone acetonide
**γ-PGA**	Poly-γ-glutamic acid
**SSTIs**	Skin and soft-tissue infections
**MRSA**	*Staphylococcus aureus*
**CP**	Chitosan-polyethyleneimine
**HPV**	Human papillomavirus
**L-PLA**	Poly-lactic-acid
**CMC**	Carboxymethyl-cellulose sodium salt
**PAH**	Primary axillary hyperhidrosis
**FMR**	Fractional microneedle radiofrequency
**SST**	Superficial skin tumors
**LCC-NPs**	Lipid-coated cisplatin nanoparticles
**APC**	Antigen-presenting cells
**GMP**	Bis-(3′–5′)-cyclic dimeric guanosine monophosphate
**GM-CSF**	Granulocyte-macrophage colony-stimulating factor
**OVA**	Ovalbumin
**TLR3**	Toll-like receptor 3
**Poly(I:C)**	Polyinosinic acid: Polycytidylic acid

### Hollow microneedles

Different from solid microneedle with a solid structure, hollow microneedle has a lumen or internal pore with a diameter approximately in the range of 50–70 μm, enabling the loading of therapeutic drugs or active solutions (Figure 2B). More content can be loaded in the lumen or internal pore of this type of microneedle than that of other microneedles. Relative to solid microneedles, the manufacturing of hollow microneedles is more complicated. The manufacturing methods include laser micromachining (Davis et al., 2005), integrated photolithography technique (Luttge et al., 2007), and micromachining (Ma et al., 2006). Hollow microneedles have been fabricated out of various materials, including silicon (Gardeniers et al., 2003), glass (Wang et al., 2006), polymers (Miller et al., 2011), metals (Lee et al., 2010). Hollow microneedles are suitable for the delivery of a variety of active molecules with relatively high drug loading capacity and accurate administration doses. However, the disadvantages include low mechanical strength and possibility of clogging and infection due to repeated application.

### Dissolving microneedles

The dissolving microneedles are typically made of water-soluble biodegradable materials, and the drug can be packaged into biodegradable materials (Figure 2C). After inserting the microneedles into the skin, the biodegradable materials dissolve and release the payload drugs (Martanto et al., 2004). Maltose (Zhang Y. et al., 2018), carboxymethylcellulose (Park and Kim, 2017), hyaluronic acid (HA) (Zhu J. et al., 2019), and other degradable materials are often used to fabricate dissolving microneedles. In contrast to other types of microneedles, dissolving microneedles are easily manufactured, and the manufacturing techniques mainly include photopolymerization (Sullivan et al., 2008), drawing photolithography (Lee and Jung, 2012), and microstructure (Shim et al., 2018). Good biocompatibility and solubility of dissolving microneedles have significantly improved patient compliance and provided new options for long-term treatment (Ita, 2015). Owing to these advantages, a variety of dissolving microneedles are being extensively investigated for the treatment of various diseases, such as cancer (Wang et al., 2016), diabetes (Zhang et al., 2019), alopecia (Cao et al., 2021), and other skin diseases (Yang et al., 2013; Du et al., 2019; Nawrocki and Cha, 2019; Song et al., 2020). However, the mechanical strength of dissolving microneedles is usually not high enough (Ita, 2017), which may influence their ability to penetrate the skin. This may inhibit complete penetration of the drugs, resulting in waste of drugs and reduced therapeutic outcomes (Lau et al., 2017).

### Coated microneedles

Coated microneedles refer to solid microneedle coated with therapeutic contents. Dipping and spraying are the two commonly used coating methods (Figure 2D). In the dipping method, the microneedle is immersed in a target coating solution to load the active content (Kapoor et al., 2020). In the spraying method, atomizer (McGrath et al., 2011) or gas jet injection (Kim et al., 2018) is applied to cover the surface of the microneedle with active ingredients. The former method can position the coating on the surface of the microneedle or only on the tip of the microneedle without polluting the base substrate. However, the latter may contaminate the substrate during the manufacturing process due to inaccurate spraying. Small-molecule drugs such as lidocaine (Ita, 2018), pilocarpine (Jiang et al., 2007), and fluorescein (Baek et al., 2017) as well as macromolecules, such as insulin (Halder et al., 2020), parathyroid hormone (1–34) (Naito et al., 2018), and hepatitis B surface antigen (Na et al., 2020) have been successfully loaded with coated microneedles.

## Penetration mechanics of microneedles

**FIGURE 3:**
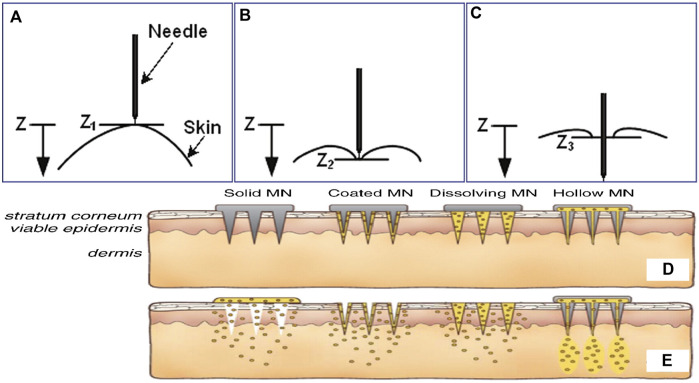
The relationship between needle and skin at different stages of insertion: **(A)** pre-puncture, **(B)** puncture, and **(C)** post-puncture. The microneedles are applied to the skin **(D)**, and then used for drug delivery **(E)**. Copyright 2017, Elsevier.

Previous studies have primarily focused on the manufacturing technologies of microneedles and their drug loading capabilities; penetration mechanics of microneedles have been often ignored. The delivery of accumulated drugs is proportional to the surface area or lumen value of microneedles, and surface area is related to the length of microneedles. Since skin is elastic, certain resistance is encountered during insertion of a microneedle (Figure 3). When the protrusion of the microneedle is too long or not strong enough, it may break during insertion into the skin (Al-Qallaf and Das, 2008). The injury induced by physical stimulation during penetration is another necessary consideration. Generally, microneedle with a length of 50–200 µm is painless. However, when the length exceeds a certain threshold, it will prick the skin, and the penetration may be unpleasant (Gill et al., 2008; Arya et al., 2017). The longer the microneedle, the higher the pain. Additionally, the geometric shape of the microneedle is very important to the insertion and penetration for drug delivery (Davis et al., 2004). If the ratio of the breaking force and insertion force is greater than 1, the microneedle can be inserted into the skin but not fractured. Thus, for maximum safety margin, the use of microneedles with small tip radius has been suggested to promote insertion and sufficient thickness to provide strength.

## Drug delivery with microneedles to treat cutaneous diseases

### Psoriasis

Psoriasis is a chronic inflammatory skin disease associated with a variety of complications that significantly lower the quality of life of patients. Approximately 125 million individuals have been diagnosed with psoriasis globally (Michalek et al., 2017; Sabri et al., 2019). The pathogenesis of psoriasis is complex and has not been fully elucidated. For patients with mild psoriasis, external drugs are still the primary form of treatment. For patients with plaque psoriasis, biologics that inhibit the production of TNF-α, p40IL-12/23, p19IL-23, and IL-17 are approved for efficient treatment (Armstrong and Read, 2020). However, these treatments can only alleviate symptoms, and no cure is known for psoriasis. Moreover, these treatments are often associated with serious side effects that worsen patient compliance (Kamata and Tada, 2018; Lockwood et al., 2018).

**FIGURE 4:**
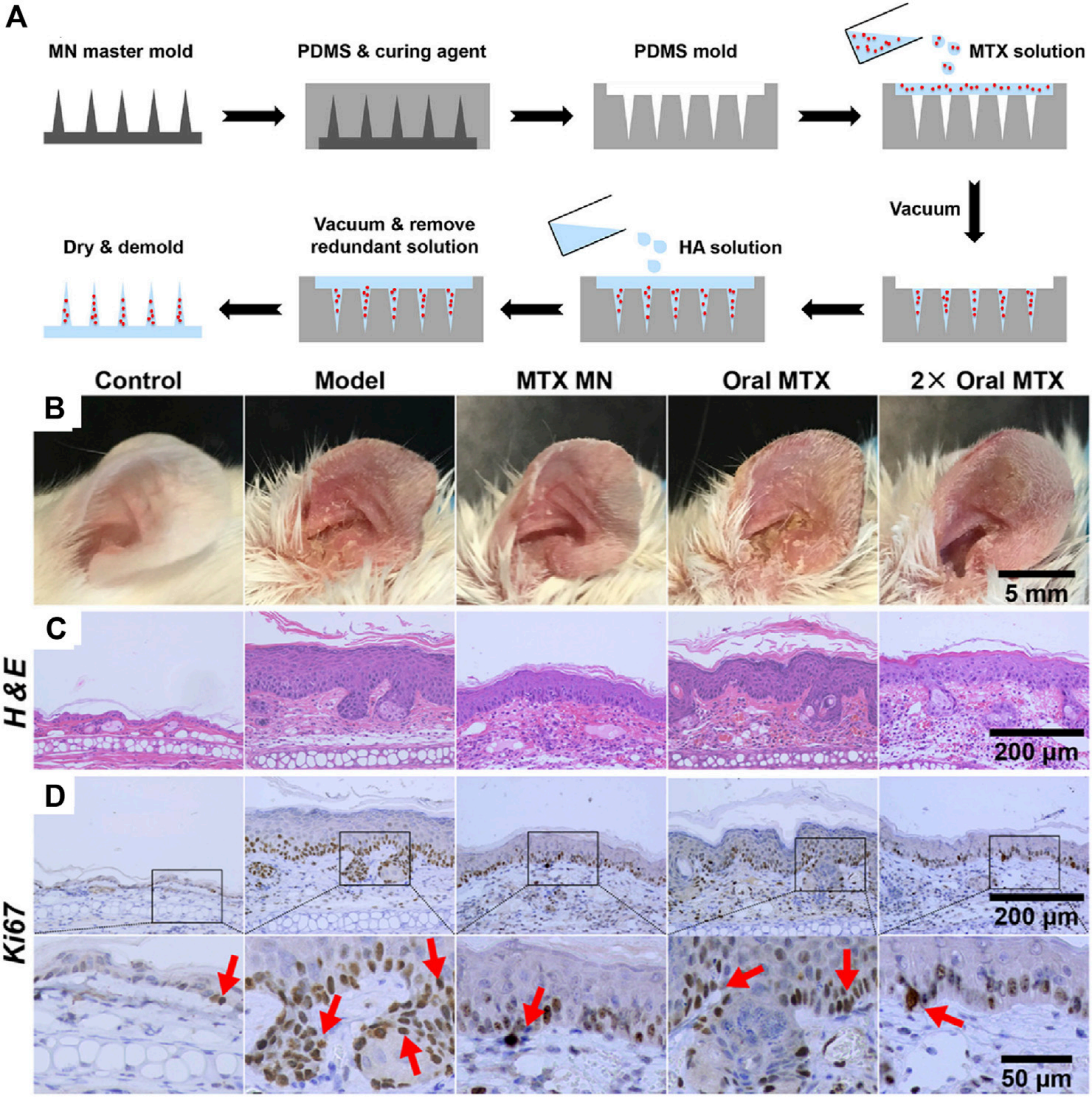
**(A)** Preparation of MTX-loaded dissoluble microneedles. Comparison of orally administered MTX and MTX loaded microneedles with transdermal administration on **(B)** left ear lesions, **(C)** H&E staining and **(D)** Ki67 IHC staining of skin sections (red arrows). Copyright 2019, American Chemical Society.

Methotrexate (MTX) is a widely used drug that could be administered *via* injection or oral routes to treat several types of cancers or to control severe psoriasis (Jolivet et al., 1983; Roenigk et al., 1988). However, adverse effects involving bone marrow inhibition, nausea, vomiting, anemia, and platelet reduction, limit its application in long-term treatment. A dissolved microneedle patch loaded with HA-encapsulated MTX for topical treatment of psoriasis was developed (Figure 4A) (Du et al., 2019). Benefiting from the highly hydrophilic HA, microneedles can dissolve in 10 min after insertion into the skin, accompanied by a triggered release of MTX for therapy. The transdermal drug release strategy involving dissolving microneedles avoids drug degradation in the gastrointestinal tract and first metabolism in the liver. MTX-loaded microneedles demonstrate higher effectiveness and reduced side effects than orally administered MTX. In addition, microneedles can also encapsulate macromolecular drugs. Dissolving microneedles containing non-soluble cyclosporin A (CyA MN) with high molecular weight for transdermal delivery of CyA were designed for the treatment of psoriasis (Jeong et al., 2018). Compared to that observed with oral routes, the pharmacokinetic behavior of CyA MN with transdermal delivery has been remarkably improved for sustained release of CyA during the dissolution of MN, with improved safety profile of CyA.

### Atopic dermatitis

Atopic dermatitis (AD) is a common chronic inflammatory skin disease, which is a result of skin barrier dysfunction and dysregulation of immune dispensing (Kim et al., 2019). Currently, cortico-steroids are the most widely used drugs to treat AD; however, long-term usage of corticosteroids may result in skin atrophy, local burning, or itching (Torrelo, 2017). Integration of corticosteroids within dissolved microneedles could concentrate their therapeutic function for better treatment outcomes, thereby decreasing the possible side effects. For instance, triamcinolone acetonide (TA) was encapsulated in dissolving microneedles, which showed a better therapeutic outcome for the high drug loading dosage (Jang et al., 2021).

**FIGURE 5:**
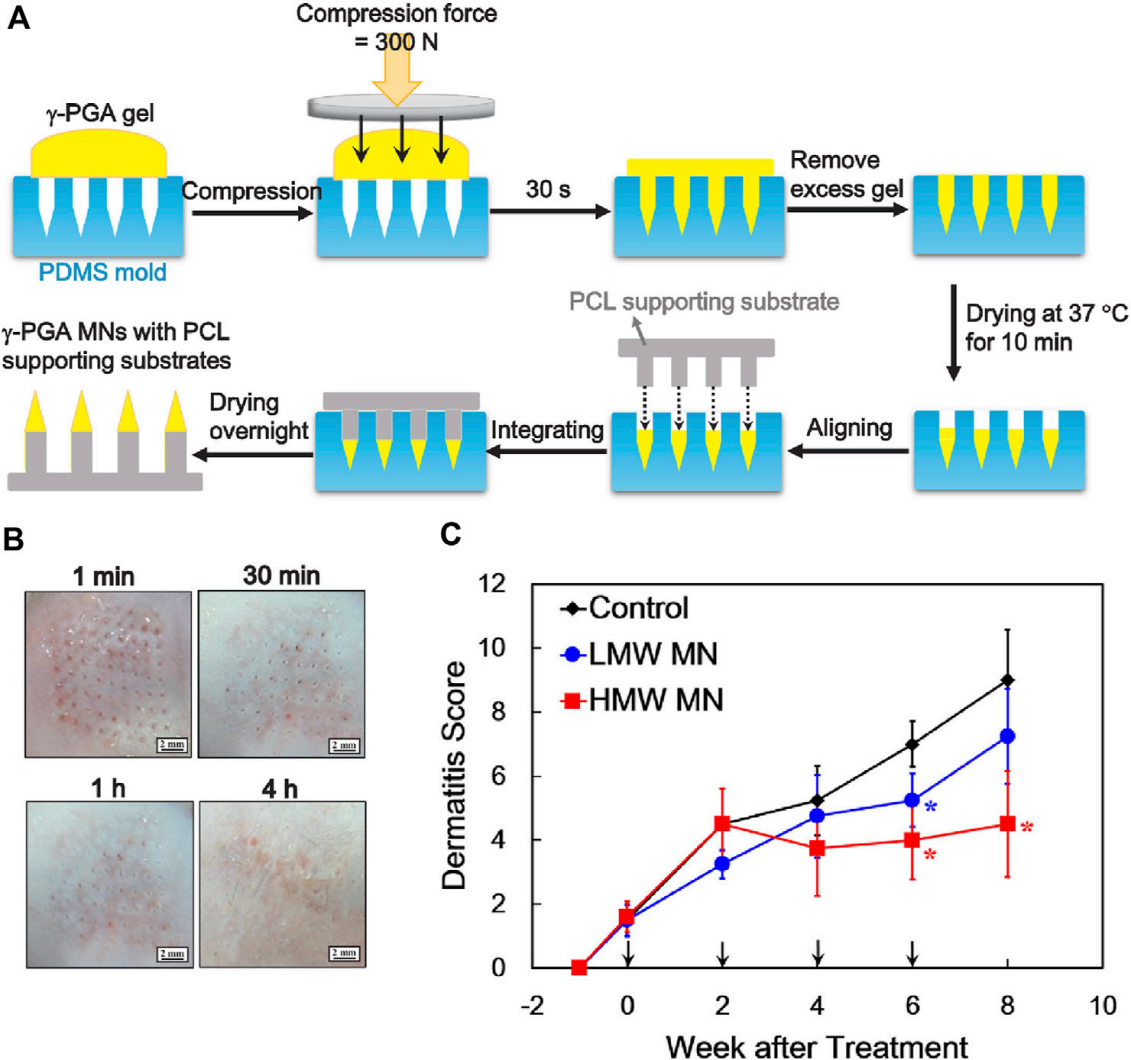
**(A)** Preparation of soluble high-molecule-wight poly-γ-PGA microneedles. **(B)** Poly-γ-PGA microneedles performing therapeutic effect within 4 h after transdermal administration. **(C)** Poly-γ-PGA microneedles could be retained in the skin for 6 days for sustained therapeutic effect. Copyright 2020, Elsevier.

In addition, previous study showed that oral administration of poly-γ-glutamic acid (γ-PGA) could activate dendritic cells (DCs) and induce production of IL-12, which decelerated AD (Sung et al., 2005; Lee et al., 2014). To avoid degradation or destruction of γ-PGA with high molecular weight in the gastrointestinal tract after oral administration, a soluble poly-γ-PGA microneedle was designed for transdermal penetration to DC-rich skin layers for efficient modulation of immune responses (Figure 5A) (Chen et al., 2020). After treatment with high molecular weight poly-γ-PGA microneedle, the skin of AD mice could quickly restore its barrier function within 4 h without any apparent skin irritation (Figure 5B). This method demonstrated a better therapeutic outcome than low molecular weight poly-γ-PGA microneedle, owing to the transdermal capacity of microneedles and high molecular weight of poly-γ-PGA could retain in the skin for 6 days for effective treatment of AD (Figure 5C).

### Skin and soft tissue infection

Skin and soft-tissue infections (SSTIs) mainly involve simple or complicated (necrotizing or non-necrotizing) infections of the epidermis, luminous membrane, subcutaneous tissue, and muscles. *Staphylococcus aureus* (MRSA), fungi, β-hemolytic *streptococcus*, or *Escherichia coli* are common pathogens implicated in SSTI. Complicated SSTI (CSSTI) is a serious infectious disease involving complications such as sepsis, which requiring surgical intervention or drug treatment (Dryden, 2010; Leong et al., 2018). Broad-spectrum antibiotics and surgical drainage are commonly applied to treat SSTIs; however, some strains showed resistance to a variety of antibiotics (Dryden, 2010).

**FIGURE 6:**
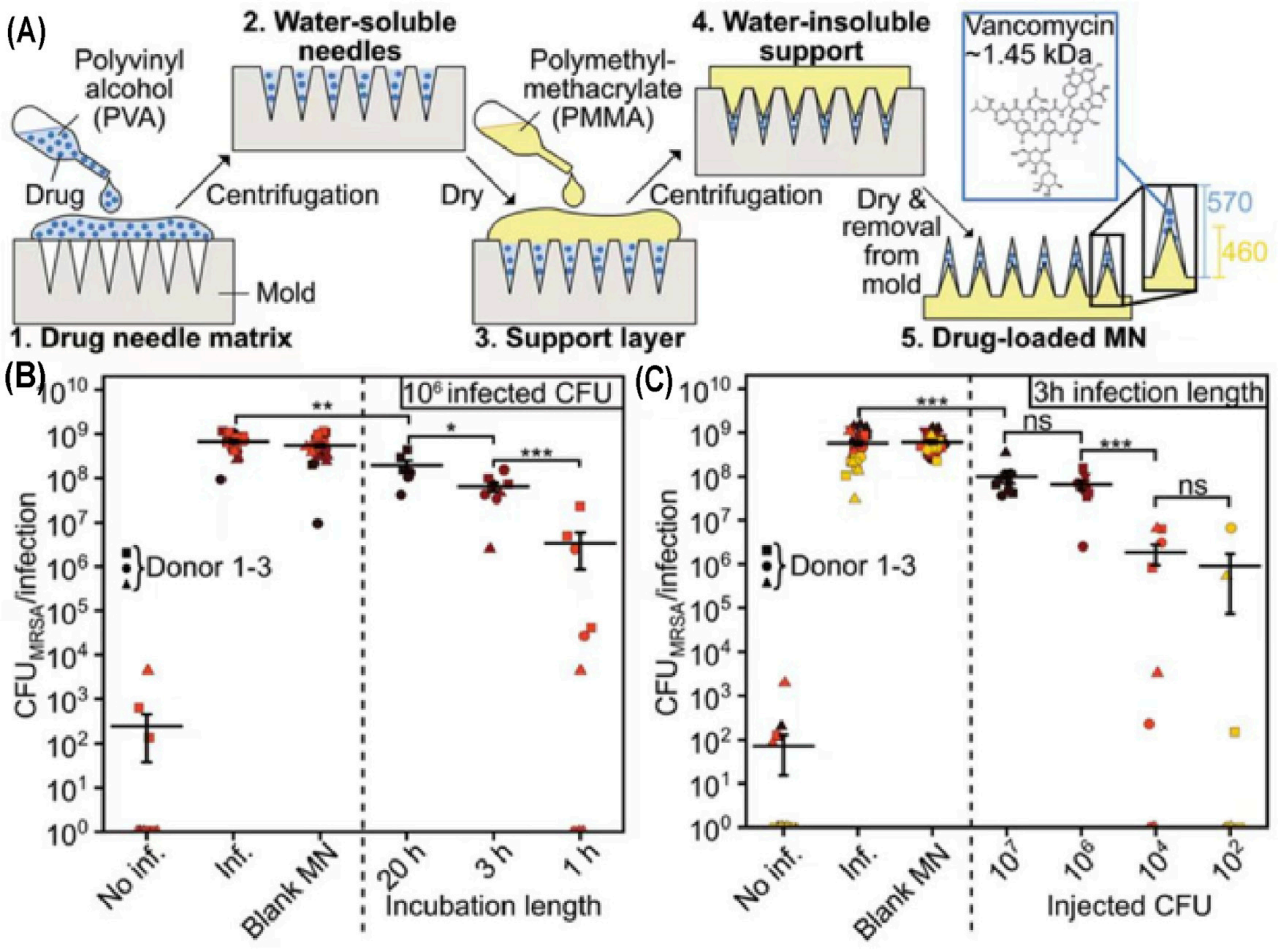
**(A)** Manufacturing process of VAN-loaded microneedle arrays. Application of MN arrays at same CFU counts **(B)** or same infection length **(C)**. Copyright 2021, John Wiley and Sons.

Therapeutic loaded transdermal microneedles can potentially mitigate these challenges (Jamaledin et al., 2020). Intravenous injection of the glycopeptide antibiotic vancomycin (VAN) was usually used to treat SSTIs caused by MRSA; however, its therapeutic efficiency was limited due to low delivery concentrations of VAN in the pathogen infected skin. Topical administration of VAN is ineffective as its high molecular weight hinders skin permeation. Sotiriou and others developed a VAN located microneedle array in the water-soluble needle tips (Figure 6A) (Ziesmer et al., 2021). The VAN-loaded microneedles could penetrate the thawed porcine and fresh human skin, triggering sustained release of a high dosage of loaded VAN within 24 h, thereby efficiently restraining MRSA (Figures 6B,C). In addition, skin fungal infection affects 20–25% global population (Havlickova et al., 2008). Fungal infections are more difficult to treat than bacterial infections owing to the presence of multiple organelles and thick and rigid cell walls that are resistant to lysis by therapeutics and innate immune response (Netea et al., 2008). To efficiently treat skin fungal infection, amphotericin B and antimicrobial chitosan-polyethyleneimine (CP) copolymer were integrated into microneedles. In addition to the absence of resistance against CP observed in pathogens, the biodegradable CP would also allow for sustained release of amphotericin B for continuous treatment after penetration into the skin, thereby facilitating synergistic effects of the antifungal polymer and amphotericin B (Zan et al., 2019). Small molecule drugs, such as doxycycline for *Staphylococcus aureus* and *Pseudomonas aeruginosa* (Permana et al., 2020), and carvacrol for multidrug-resistant bacteria (Mir et al., 2020) have also been loaded into microneedles for better therapeutic outcomes.

In addition to the aforementioned traditional drugs, living bacteria could also be encapsulated within microneedles. Bacillus subtilis (*B. subtilis*)-encapsulated microneedles could prevent the escaping of *B. subtilis* and continuously secrete various antifungal agents, which can directly bind to fungal cell surface-associated proteins and destruct the fungal cell membranes without inducing drug resistance (Wang F. et al., 2020).

### Plantar warts

Plantar warts are a common skin disease observed at the plantar site caused by human papillomavirus (HPV) (Bacelieri and Johnson, 2005; Konicke and Olasz, 2016). Several methods, such as salicylic acid, cryotherapy, surgical resection, and laser can be used for plantar warts treatment (Bacelieri and Johnson, 2005). However, these treatments have limitations, such as pain induced by surgical intervention and continuous medication (Clifton et al., 2003; Saitta et al., 2008), and no single therapy can cure plantar warts completely.

**FIGURE 7:**
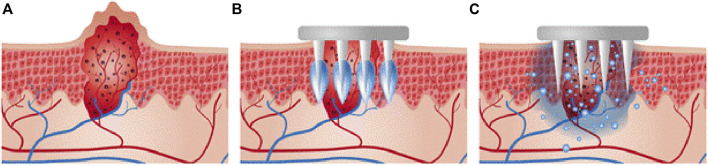
**(A)** Schematic diagram of a cross-section of warts. **(B)** Insertion of the bleomycin-coated microneedle. **(C)** Transdermal delivery of bleomycin into warts. Copyright 2017, Springer Nature.

Bleomycin is a traditional drug used for the treatment of warts. Intralesional bleomycin has been demonstrated as an effective choice for refractory warts, but with disadvantages of scarring induction, pigmentation, and nail damage (Bik et al., 2020). To relieve these side effects, microneedles with targeted spraying of bleomycin have demonstrated higher clearance and low pain compared to intralesional bleomycin (Saitta et al., 2008; Al-Naggar et al., 2019). In another study, bleomycin was directly coated on the tips of poly-lactic-acid (L-PLA) microneedles, which were fabricated by a molding process (Lee et al., 2017). Carboxymethyl-cellulose sodium salt (CMC) was used as a thickening agent on the tips of microneedles for loading more bleomycin (Figure 7). Assisted with CMC, bleomycin could be coated on the tips of microneedles with a high dosage (518 µg) and demonstrate high skin tolerance. More than 80% of coated bleomycin can be delivered into the skin, and a more concentrated bleomycin delivered into the subepidermis layer, which leading to improved therapeutic outcomes compared to those observed with intralesional injection.

### Axillary hyperhidrosis

Primary axillary hyperhidrosis (PAH) is characterized by an increased amount of sweat production, specifically in the axilla, which can lead to social embarrassment, emotional and occupational distress (Lakraj et al., 2013; Hamm, 2014). Current treatments mainly include topical aluminum salts, iontophoresis, Botox therapy, and surgical sympathectomy (Hoorens and Ongenae, 2012). However, these treatment modalities are far from satisfactory (Doft et al., 2012). For instance, the disadvantage of using botulinum toxin is that about 20 painful intradermal injections must be repeated every 3 months.

To improve the administration procedure and therapeutic efficiency, Coulman and others reported a pocketed microneedle for delivery of liquid state botulinum toxin A formulation into human skin (Torrisi et al., 2013), demonstrating a potent therapeutic route for PAH. In addition, transdermal delivery of radiofrequency by fractional microneedle radiofrequency (FMR) could also present an alternative treatment strategy. As an effective treatment method for PAH, FMR could damage eccrine glands *via* thermolysis at the interface of the deep dermis and subcutis after transdermal penetration of FMR while minimizing side effects on the surrounding tissues (Kim et al., 2013; Zhang M. et al., 2018).

### Superficial tumors

Superficial skin tumors (SST) are the most common tumors observed in human, mainly classified as hemangioma, actinic keratosis, and squamous cell carcinoma, which are caused by excessive proliferation and mutation of skin cells (Lomas et al., 2012; Grossman et al., 2018). Most traditional chemotherapeutic agents used for superficial tumors, such as docetaxel, cisplatin, and doxorubicin, are generally administered intravenously (Dalbagni, 2007; Gao et al., 2017). However, systemic administration of chemotherapeutics can induce many adverse effects, such as bone marrow suppression, neurotoxicity, and adverse gastrointestinal reactions (Heidary et al., 2008), which impair therapeutic outcomes and are very uncomfortable for patients.

**FIGURE 8:**
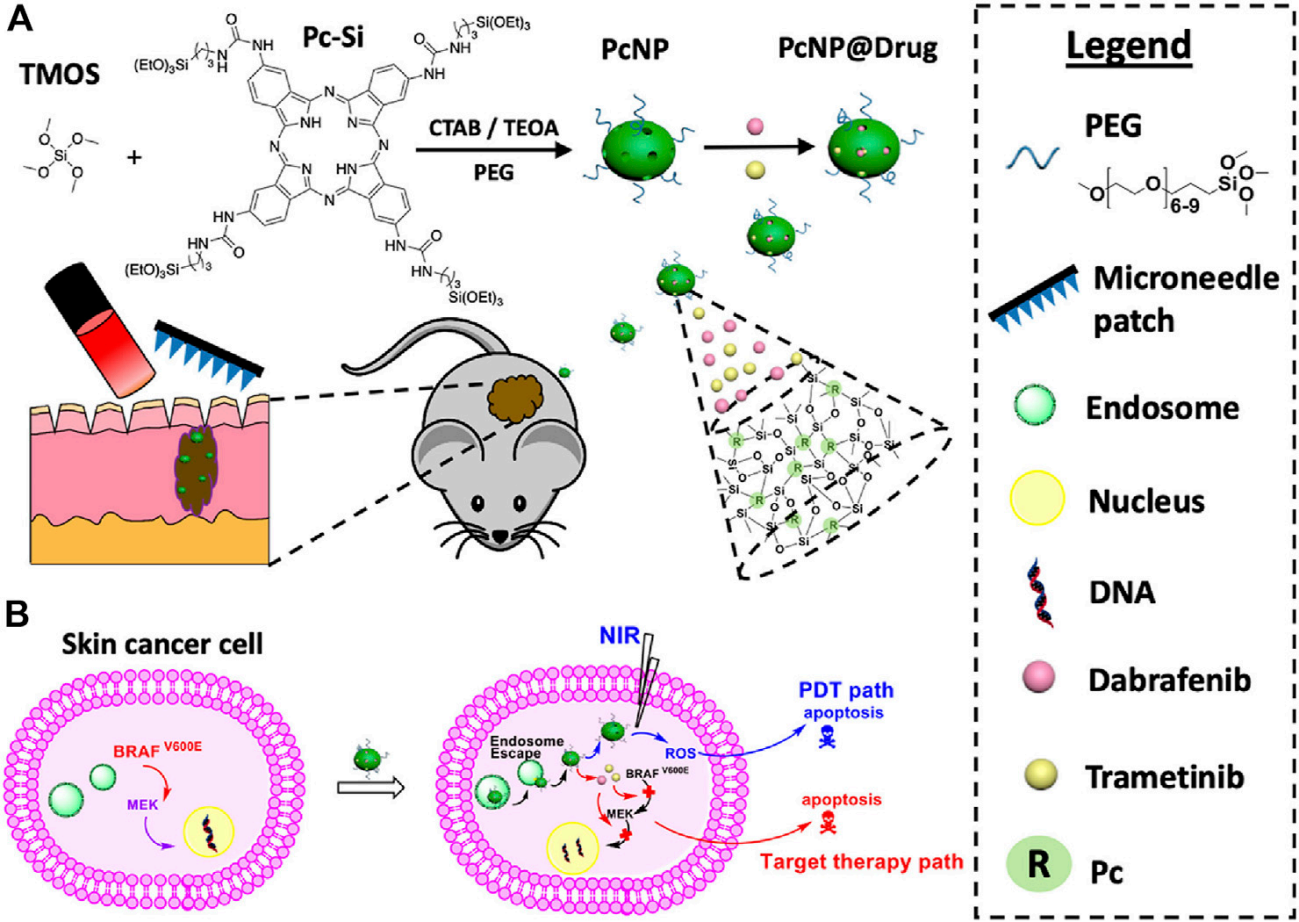
**(A)** Schematic illustration of co-loaded photosensitizers and small-molecule inhibitors into microneedles for synergistic therapy of deep-seated melanoma. **(B)** Antitumor effects for these transdermally delivered drugs. Copyright 2018, American Chemical Society.

Compared to systemic administration of chemotherapeutics, microneedle-mediated drug delivery systems demonstrate natural advantages for treating superficial skin tumors owing to the precise administration, local drug release, good biocompatibility, and less pain. The topical administration of chemotherapeutic encapsulated microneedles can greatly improve the targeting efficiency and reduce adverse effects. For example, cisplatin, as a first-line chemotherapeutic agent, may exhibit side effects of nephrotoxicity, neurotoxicity, ototoxicity, electrolyte disturbance, and hemolytic anemia (Loehrer And Einhorn, 1984; Minami et al., 2004; Barabas et al., 2008). To avoid these toxic effects, lipid-coated cisplatin nanoparticles (LCC-NPs) were formulated using tumor-targeting pH-responsive lipid nanoparticles and embedded into dissolvable microneedles for transdermal administration (Lan et al., 2018). After insertion into the skin, the nanoparticles can be locally delivered through the stratum corneum. *In vivo* study demonstrated that microneedle arrays significantly increased therapeutic results with reduced systemic toxicity and side effects. Microneedles are also used to synergistically treat superficial tumors with phototherapy (Chen et al., 2016; Dong et al., 2018; Tham et al., 2018; Song et al., 2020). Zhao and others developed a microneedle-assisted topical delivery system encapsulated with active mesoporous organosilica nanoparticles preconjugated with a photosensitizer (phthalocyanine) and simultaneously coated with small molecule inhibitors (dabrafenib and trametinib) (Figure 8A) (Tham et al., 2018). Microneedles could facilitate the penetration of active mesoporous organosilica nanoparticles to reach deep-seated melanoma sites. Assisted with NIR irradiation (Figure 8B), the drug-loaded microneedle platform showed a synergistic killing effect on skin cancer cells with reactive oxygen species induced by the photosensitizer and caspase-activated apoptosis for small-molecule inhibitors without systemic toxicity.

## Other applications

### Microneedle-mediated delivery of vaccines

Vaccination is considered one of the most important methods for protection against infectious diseases. As the most recent example, the coronavirus disease 2019 (COVID-19) pandemic caused by the novel Coronavirus strain SARS-CoV-2 is still raging (Cullen et al., 2020; Daniel, 2020; Donthu and Gustafsson, 2020; Korbel and Stegle, 2020; Van Bavel et al., 2020). Vaccines play an important role to reduce the infection spread and serious symptoms (Wang J. et al., 2020; Malik et al., 2020). Vaccine administration mainly includes traditional parenteral and mucosal routes, novel needle-free injection and adjuvant formulations (Zhang et al., 2015). The needle-based delivery system functions as the standard intradermal injection technique that involves the insertion of the tip into the skin and pushing the contents within a plastic 1 ml disposable syringe (Lambert and Laurent, 2008). For avoiding disadvantages of needle-based administrations, vaccine delivery systems facilitated by needle-free injection, liquid jet injectors, ballistic injectors, and microneedle injection have been developed (Giudice and Campbell, 2006; Kis et al., 2012; Hossain et al., 2020). Different delivery methods have different immune efficiency attributed to the priming of immune cells and subsequent immune response.

**FIGURE 9:**
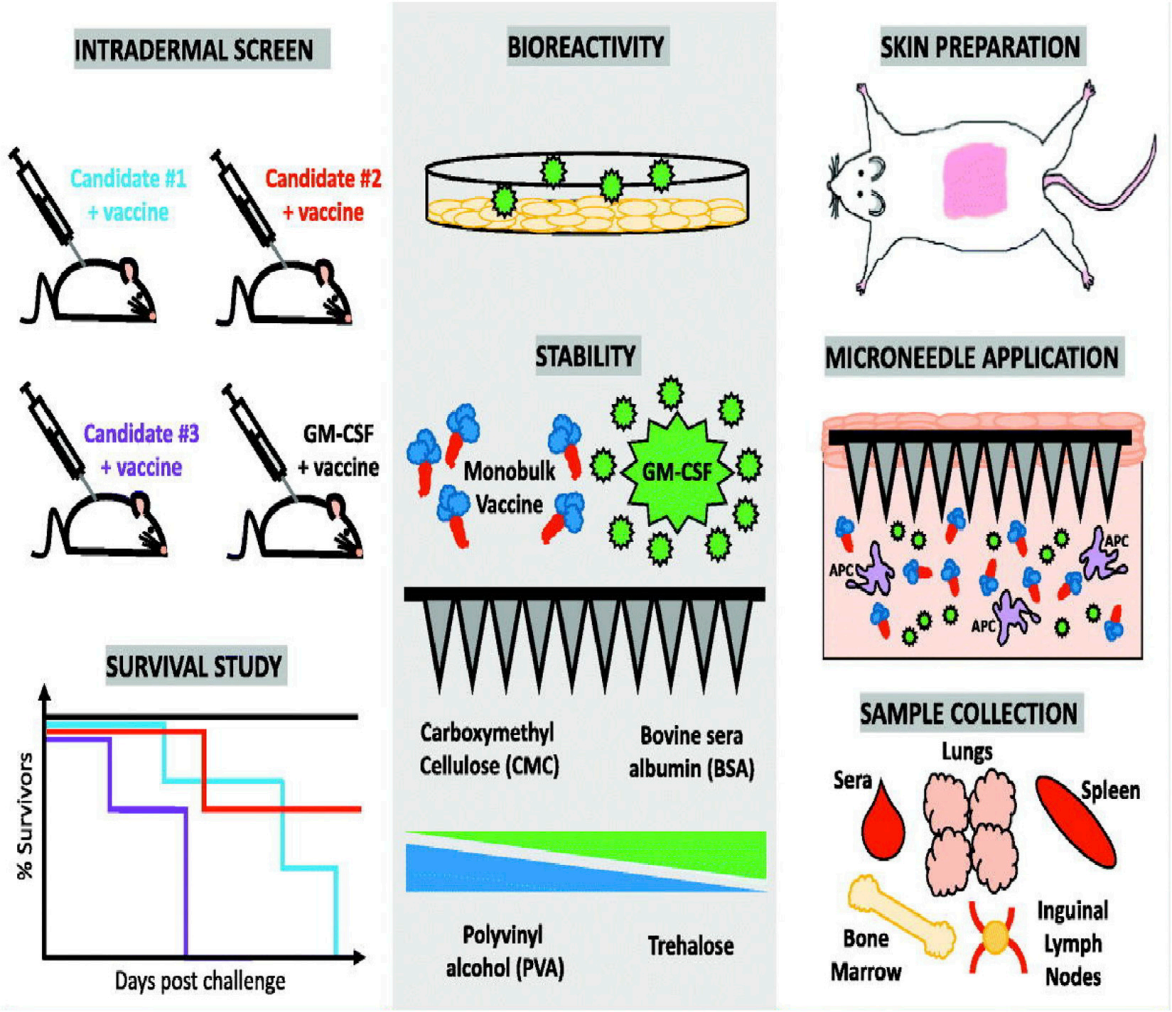
Preparation of granulocyte-macrophage colony-stimulating factor (GM-CSF) loaded microneedles and immune response after transdermal delivery performed using GM-CSF-loaded microneedles. Copyright 2018, Elsevier.

Convenient and favorable delivery system is very important for optimal potency of vaccines. Microneedle-based vaccine delivery platform has simplified vaccine distribution, improved patient compliance, and targeted vaccine delivery in the skin to activate antigen-presenting cells (APC) (Van der Maaden et al., 2012; Ma and Wu, 2017; Rodgers et al., 2018; Li Z. et al., 2020). In addition, compared with needle-based muscle injection, the immune memory reaction triggered by microneedle-based administration is enhanced with the continuous release of the vaccine loaded within the cavity of microneedles (Kim et al., 2010). For example, vaccines delivered with a hollow microneedle demonstrated enhanced immunity than subcutaneous injection (Ogai et al., 2018) as the accurately delivery of vaccine into the upper portion of the dermis, inducing enhanced immune responses. Owing to these advantages, microneedle-based vaccination has been widely studied for immunization against influenza (Rouphael et al., 2017; Zhu et al., 2018; Shin et al., 2020). Bis-(3′–5′)-cyclic dimeric guanosine monophosphate (GMP, a bacterial second messenger and stimulator of interferon gene agonist), which is a suitable adjuvant, and influenza vaccine were loaded into the microneedle and then transdermally delivered to the skin of the mice. The immunogenicity and protective effects after inoculation were assessed. Mice immunized with 2 μg of GMP and influenza viruses showed a higher level of IgG and systemic immune response than mice immunized alone. This finding demonstrated that simultaneous delivery of influenza vaccines and GMP adjuvant with microneedles could increase the immune efficiency of the vaccine. Granulocyte-macrophage colony-stimulating factor (GM-CSF) can also strengthen the antibody reaction of the influenza vaccine after co-loading into a dissolvable microneedle patch (Littauer et al., 2018), which improved the protective effects of the vaccine by promoting the proliferation of vaccine specific T cells (Figure 9).

Other vaccines can also be loaded with microneedles for efficient vaccination. A soluble microneedle manufactured using carboxymethyl cellulose and trehalose was used to load adenovirus vaccine (Ad5. OVA) together with toll-like receptor 3 (TLR3) agonist polyinosinic acid: polycytidylic acid [Poly (I:C)] (Erdos et al., 2020). After insertion into the skin, the microneedles can dissolve and release loaded adenovirus and Poly (I:C), resulting in overexpression of the OVA transgene and corresponding OVA mRNA. In addition, the Pastis toxin (PT) vaccine delivered by microneedles (Zhu D. D. et al., 2019), which can be successfully inserted into mice at a depth of 330 μm, which is abundant with immune cells. Compared to direct subcutaneous injection, the antigen release duration for mi-croneedle vaccination was longer, and less amount of PT antigen could elicit strong immune re-sponses. This vaccination route presented significant advantages over traditional subcutaneous in-jections.

### Aesthetic medicine

Microneedles also present promising application in aesthetic medicine (Cachafeiro et al., 2016; Iriarte et al., 2017; Sitohang et al., 2021). Microneedles exert effects mainly by piercing the epidermal skin and enhancing the release of loaded active ingredients, which can lead to a reduction in skin scars, pigmentation, and wrinkles (Choy and Prausnitz, 2011). Dermaroller ^®^ (Dermaroller GmbH, Wolfenbüttel, Germany) is a commonly used microneedle device, which contains 192 needles, each with a length of 0.5–3 mm and base diameter of 0.1–0.25 mm. They are arranged in 24 circular arrays. By scrolling, the microneedles are inserted into the epidermis and papillary leather, producing hundreds of mini small holes, followed by topical release of active ingredients (Shin et al., 2012; Shukla and Gold, 2021). Compared with traditional methods, microneedles can increase the delivery efficiency of medicines with higher safety and effectiveness (Cachafeiro et al., 2016), as the micropores generated by the microneedle insertion can be healed within a few hours (Aung et al., 2020). Furthermore, minoxidil-loaded microneedles can stimulate hair follicle stem cells and activate growth factors in the dermal nurtle, which can promote hair growth in alopecia (Faghihi et al., 2021). Owing to these advantages, many clinical trials of microneedle-based products are being conducted for aesthetic medicine applications (Hoesly et al., 2012; Min et al., 2015; Kwon et al., 2017; Biesman et al., 2019).

## Summary

In the past 10 years, remarkable advances have been made in microneedle-based drug delivery systems. Many microneedle-based products have been patented and entered the clinical trial stage, especially in aesthetic medicine. However, most microneedle-based drug delivery systems are still needed to improve for better applications. For example, microneedles may induce a slightly short skin stimulus, inducing local spotted erythema and edema, the holes after penetration with microneedles may also cause skin infections. Microneedle-based products need more extensive clinical investigation for improved compatibility and further extensive application. With the development of biocompatible polymers, microneedle systems with good biocompatibility and solubility are expected to be designed. At that time, microneedle will have a higher impact in the clinic and will further improve human health through a variety of drug delivery methods.
